# Estimating the Frequency of Suicide Related to Driving Cessation Among Older Adults: A Data Science Approach

**DOI:** 10.1093/geroni/igaa057.1506

**Published:** 2020-12-16

**Authors:** Tomohiro Ko, Viktoryia Kalesnikava, Briana Mezuk

**Affiliations:** 1 University of Michigan School of Public Health, Ann Arbor, Michigan, United States; 2 University of Michigan, Ann Arbor, Michigan, United States

## Abstract

Up to 35% of older adults in the United States engage in partial to complete driving cessation. Suicide risk is highest among older adults, and driving cessation can precipitate psychosocial changes linked with elevated risk of suicide, such as loss of independence, mobility, and socialization. Little is known about the association between suicide and driving cessation among older adults. We developed a supervised machine learning natural language processing algorithm to estimate the frequency of and characterize suicides associated with driving cessation from restricted-access case narratives in the National Violent Death Reporting System (NVDRS, 2003-2015) from suicides and undetermined deaths among adults 55 years and older in 27 states. Among 47,759 decedents, preliminary results identified 972 deaths associated with driving cessation, with a mean age of 71.4, 91.5% White, and 78.3% Male. 13.8% of the decedents of driving cessation-associated suicide had a crisis contributing to the suicide within 2 weeks of death. This analysis assesses the relationship between contextual geographic features such as population density and the distribution of driving cessation-associated suicides across the US. Government and clinical stakeholders should invest in public transportation infrastructure catered to older adults and implement policies to reduce the burden of driving cessation. Increased accessibility of technological innovations such as self-driving cars may also enhance quality of life for a growing older adult population.

